# Supersaturation Potential of Amorphous Active Pharmaceutical Ingredients after Long-Term Storage

**DOI:** 10.3390/molecules24152731

**Published:** 2019-07-27

**Authors:** Khadijah Edueng, Denny Mahlin, Johan Gråsjö, Olivia Nylander, Manish Thakrani, Christel A.S. Bergström

**Affiliations:** 1 Department of Pharmacy, Uppsala University, BMC P.O. Box 580, Husargatan 3, 75123 Uppsala, Sweden; 2 Kulliyyah of Pharmacy, International Islamic University Malaysia, Jalan Istana, Bandar Indera Mahkota, 25200 Kuantan Pahang, Malaysia; 3 AstraZeneca Operations, Forskargatan 18, 15185 Södertälje, Sweden; 4 Department of Pharmacy, University College London (UCL), Brunswick Square, WC1N 1AX London, UK; 5 The Swedish Drug Delivery Forum, Department of Pharmacy, Uppsala University, BMC P.O. Box 580, Husargatan 3, 75123 Uppsala, Sweden

**Keywords:** physical aging, crystallization, amorphous, supersaturation potential, crystallization kinetics, nucleation pathway, crystal growth, dissolution, solvent shift, spray-drying

## Abstract

This study explores the effect of physical aging and/or crystallization on the supersaturation potential and crystallization kinetics of amorphous active pharmaceutical ingredients (APIs). Spray-dried, fully amorphous indapamide, metolazone, glibenclamide, hydrocortisone, hydrochlorothiazide, ketoconazole, and sulfathiazole were used as model APIs. The parameters used to assess the supersaturation potential and crystallization kinetics were the maximum supersaturation concentration (C_max,app_), the area under the curve (AUC), and the crystallization rate constant (k). These were compared for freshly spray-dried and aged/crystallized samples. Aged samples were stored at 75% relative humidity for 168 days (6 months) or until they were completely crystallized, whichever came first. The solid-state changes were monitored with differential scanning calorimetry, Raman spectroscopy, and powder X-ray diffraction. Supersaturation potential and crystallization kinetics were investigated using a tenfold supersaturation ratio compared to the thermodynamic solubility using the µDISS Profiler. The physically aged indapamide and metolazone and the minimally crystallized glibenclamide and hydrocortisone did not show significant differences in their C_max,app_ and AUC when compared to the freshly spray-dried samples. Ketoconazole, with a crystalline content of 23%, reduced its C_max,app_ and AUC by 50%, with C_max,app_ being the same as the crystalline solubility. The AUC of aged metolazone, one of the two compounds that remained completely amorphous after storage, significantly improved as the crystallization kinetics significantly decreased. Glibenclamide improved the most in its supersaturation potential from amorphization. The study also revealed that, besides solid-state crystallization during storage, crystallization during dissolution and its corresponding pathway may significantly compromise the supersaturation potential of fully amorphous APIs.

## 1. Introduction

Oral administration of medicines is the most commonly used route owing to its convenience, good patient compliance, relatively low cost, less stringent manufacturing requirements, and wide selection of dosage form design [1]. For solid dosage form design, a crystalline form of the active pharmaceutical ingredient (API) is often preferred due to its excellent physical and chemical stability [2]. In order for a solid crystalline API to dissolve in aqueous media, three major processes have to take place. First, the crystal lattice of the crystalline API has to be disrupted so that individual molecules can be separated and be solvated in the subsequent step. At the same time, hydrogen bonds binding water molecules together need to be broken to accommodate the solute (API) molecules [3]. Limited aqueous solubility of an API may be caused by high lattice energy, poor hydration, or a combination of the two [4].

These energy barriers do not impose any substantial problem on the dissolution of highly soluble APIs (Biopharmaceutics Classification System, BCS Classes I and III), but they may significantly hinder the solubility and dissolution of poorly soluble APIs belonging to BCS Class II or IV. However, this particular classification also considers dose in the determination of the solubility class, meaning that high dose with a moderate solubility (e.g., 100 µg/mL) will be classified as poorly soluble. Usually, poorly water-soluble APIs display slow dissolution in vivo. This results in incomplete dissolution during the transit time through the small intestine where most of the drug absorption occurs. This phenomenon eventually leads to a suboptimal pharmacological effect of the drug treatment [5]. As the number of poorly water-soluble APIs within the pharmaceutical pipeline continues to rise [6,7,8,9], formulation scientists try to develop formulations that can improve the dissolution rate (and/or solubility) of such APIs. One of the formulation strategies that has received a lot of attention within the field is amorphous formulation [10].

Unlike a crystalline API, the amorphous API is more disordered in its structure due to the lack of long-range order of the molecules. This increases the Gibbs free energy and diminishes the need to overcome the crystal lattice energy during the dissolution event. As such, the amorphous API should have superior solubility and dissolution rate than its crystalline counterpart [11,12,13,14]. However, the higher energy state is also a major pitfall for amorphous materials. The material is thermodynamically unstable, which over time results in relaxation, nucleation, and crystal growth. All these events can occur during manufacturing, handling, storage, and upon dissolution following oral administration [4,15].

Upon storage, amorphous materials can undergo two main processes that may result in loss of the amorphous form—enthalpic relaxation (especially if stored below the glass transition temperature (T_g_)) and crystallization. The enthalpic relaxation involves molecular motions towards equilibrium that contribute to physical aging of the material [15,16]. The enthalpic relaxation and recrystallization of an amorphous solid may be facilitated and accelerated in the presence of water. Water is, in this process, a potent plasticizer. The spontaneous and significant absorption of water (T_g_ = −137 °C) by amorphous solids decreases the T_g_ of the amorphous solid. The decrease in T_g_, in turn, increases the molecular mobility, allowing the molecules to rearrange themselves into the more stable long-range arrangement of the crystalline solid [17,18,19].

During storage of amorphous materials, enthalpic relaxation and recrystallization reflect molecular motions in an attempt by the solid to reach thermodynamic equilibrium and reformation of the crystal structure, respectively. These phenomena result in an aged amorphous phase or a crystalline solid, and for both of these structures, a relatively higher energy is required to break the molecular interactions than for the fresh amorphous material. Therefore, the solubility and dissolution rate advantage gained from amorphization might be compromised.

For a fully recrystallized solid, the solubility and/or dissolution rate is similar or comparable to that of its crystalline counterpart, provided that the following conditions are fulfilled: (i) it recrystallizes to the original crystal form and not to another polymorph with different solubility and dissolution profiles; and (ii) the concentration–time profile is primarily affected by the crystallization that occurs during dissolution. In theory, the physically aged and recrystallized amorphous solid should have a lower solubility and/or dissolution rate than the fresh amorphous solid, but still, little is known about the extent to which physical aging and partial crystallization of an amorphous solid affect the resulting kinetic solubility and dissolution rate. Similarly, little is known about the impact of physical aging on the rate of crystallization and precipitation of amorphous APIs during dissolution. The only available studies are either on a single model compound, amorphous solid dispersion (i.e., in the presence of a stabilizing polymer), or these two in concert [20,21]. For an amorphous system, the solubility and/or dissolution performance is often assessed on the basis of the maximum supersaturation concentration achieved (C_max_), the extent of supersaturation (area under the curve, AUC), and the crystallization rate (k). The latter is typically determined by analysis of the precipitation rate.

In this work, we therefore studied the impact of long-term physical aging (168 days, i.e., 6 months) and potential crystallization upon storage of seven amorphous APIs. The impact was assessed from measurement of (i) the apparent maximum supersaturation concentration achieved (C_max,app_); (ii) the extent of supersaturation (AUC); and (iii) the crystallization rate constant (k). These properties were measured during dissolution in aqueous buffer at pH 6.5 to simulate the pH of small intestinal fluid. The data obtained were then linked to the ageing effects and crystallization pattern of the APIs.

## 2. Results and Discussion

### 2.1. Physical Stability and Crystallization Behavior of Spray-Dried Amorphous APIs

The spray-dried amorphous APIs demonstrated different physical stability profiles when stored at 75% RH (see Table 1). In other words, the crystallization propensity varied for the seven APIs upon exposure to humidity. Two compounds (indapamide and metolazone) remained amorphous for 168 days, i.e., they were highly resistant to crystallization. The remaining five APIs started to crystallize already after one day of exposure to 75% RH. What was striking for these five was that, even though they started crystallizing around the same time, the time to complete crystallization varied tremendously. For instance, glibenclamide and hydrocortisone began to crystallize after one day of storage but were not completely crystallized when the stability study was terminated after 6 months. At the completion of the stability test, these compounds were a mixture of amorphous and crystalline solid, with approximately 94% and 89% of the mixture composition still being amorphous, respectively. In contrast, ketoconazole had a different crystallization profile compared to the other four. It started to crystallize on the first day of exposure and was completely crystallized by the seventh day. At the other end of the spectrum, hydrochlorothiazide and sulfathiazole were completely crystalline already after one day of storage at 75% RH.

On the basis of these stability and crystallization profiles, the initial crystallization propensity of our model APIs can be categorized as (i) low initially, with slow crystal growth; (ii) high initially, with slow crystal growth; or (iii) high initially, with rapid crystal growth (see Table 1). The category to which the API belongs is an indication of its propensity to initiate crystallization and the speed at which crystal growth occurs in the presence of humidity. It may be possible for some APIs to be physically stable for a considerably long time without any stabilizer or crystallization inhibitors, but others might crystallize rather quickly if such excipients are not present. Furthermore, an API with high crystallization initiation propensity is not necessarily a fast crystal grower, as exemplified by glibenclamide and hydrocortisone in our study.

### 2.2. Supersaturation Potential and Crystallization Kinetics as a Function of Physical Stability

**Figure 1:**
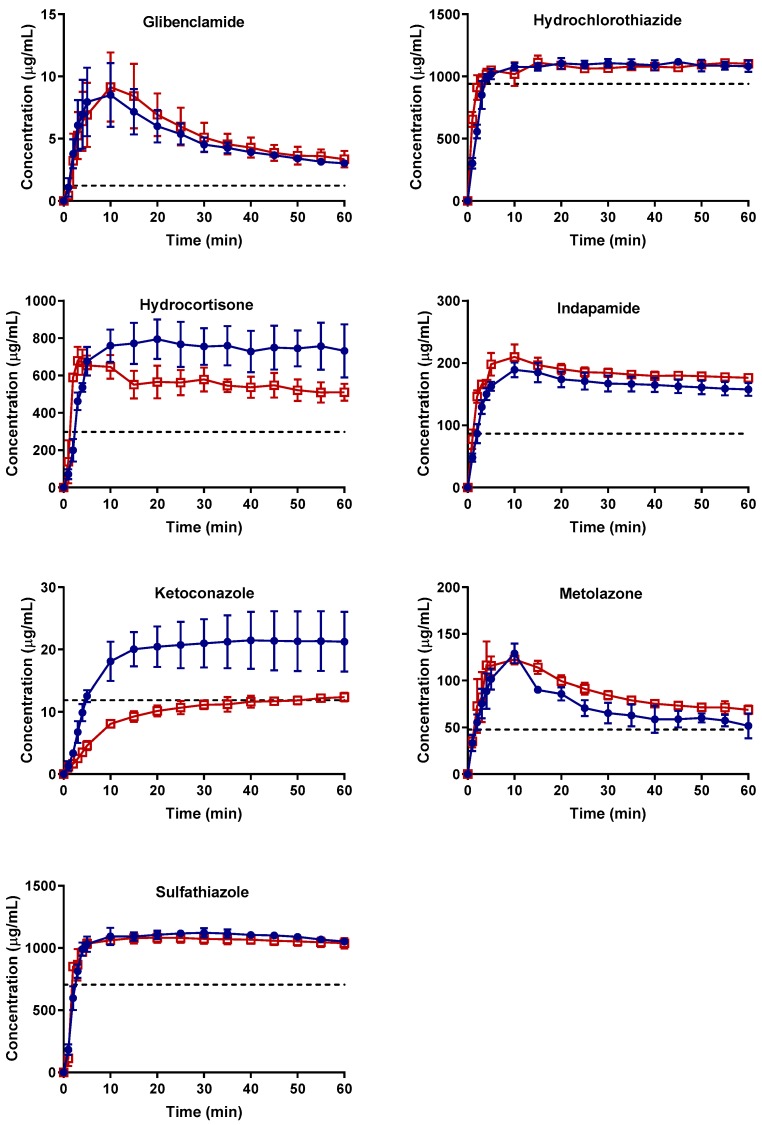
Concentration–time profiles of freshly spray-dried APIs (SD T_0_) shown as blue circles. The crystallized and/or aged spray-dried APIs (SD T_n_) are shown as red empty squares. The time point n depends on (i) the time point at which the spray-dried samples completely crystallized or (ii) the last time point of the stability study (i.e., 168 days) if crystallization was incomplete or did not happen (as indicated in Table 1). The apparent crystalline solubility of each API is shown as a black dashed line. The mean ± standard deviation values of three replicates are shown.

The concentration–time profiles of the APIs are shown in Figure 1. Overall, none of the APIs had clearly distinguishable differences in the dissolution profiles for the fresh and aged (and/or crystallized) spray-dried samples, except for ketoconazole. For this API, the dissolution of the completely crystallized form was similar within 60 min to the apparent solubility of the original crystalline form. All of the APIs exhibited some greater degree of supersaturation than their crystalline counterparts. Indapamide, metolazone, and glibenclamide displayed unstable supersaturation as evidenced by a decrease in concentration after reaching a certain C_max,app_ above the apparent crystalline solubility. In contrast, hydrocortisone, hydrochlorothiazide, ketoconazole, and sulfathiazole dissolved, forming an apparently stable supersaturation for 60 min at a concentration slightly higher than their apparent crystalline solubility. With comparable estimated crystalline contents of 6% and 11%, respectively, glibenclamide and hydrocortisone demonstrated very different supersaturation profiles. For glibenclamide, the concentration–time profiles of the fresh and aged and/or crystallized samples are practically the same. On the contrary, both hydrocortisone samples achieved a comparable C_max,app_, but the aged and/or crystallized samples showed a slight decrease in concentration over time compared to the fresh one. Nevertheless, both hydrocortisone samples ended up at concentration higher than their crystalline solubility after 60 min of dissolution.

#### 2.2.1. Apparent C_max_ and AUC Ratio of Fresh and Aged and/or Crystallized Spray-Dried Solids

**Figure 2:**
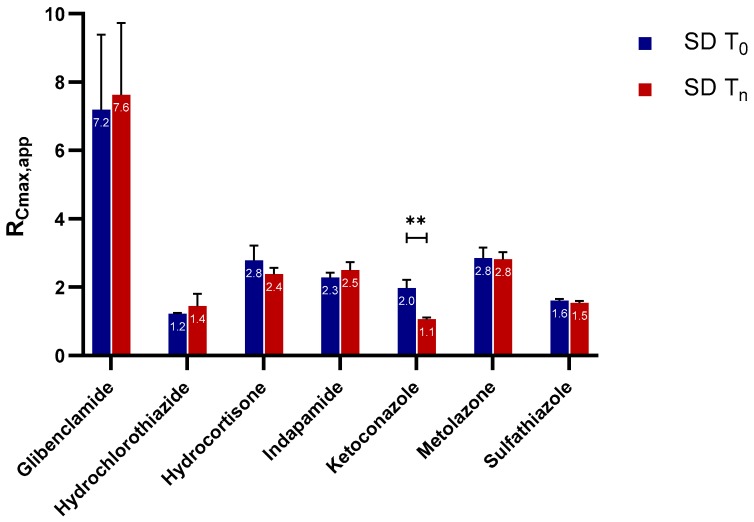
The apparent maximum supersaturation concentration (C_max_) ratios (R_Cmax,app_) of the fresh and aged and/or crystallized spray-dried APIs at tenfold supersaturation ratios. The freshly spray-dried samples are represented as SD T_0_, whereas the crystallized and/or aged samples are denoted SD T_n_ since each of the APIs crystallized at different time points (see Table 1). At a 95% confidence interval, a *p*-value of <0.05 is considered statistically significant. The mean ± standard deviation values of three replicates are shown. ** denotes *p* < 0.01.

**Figure 3:**
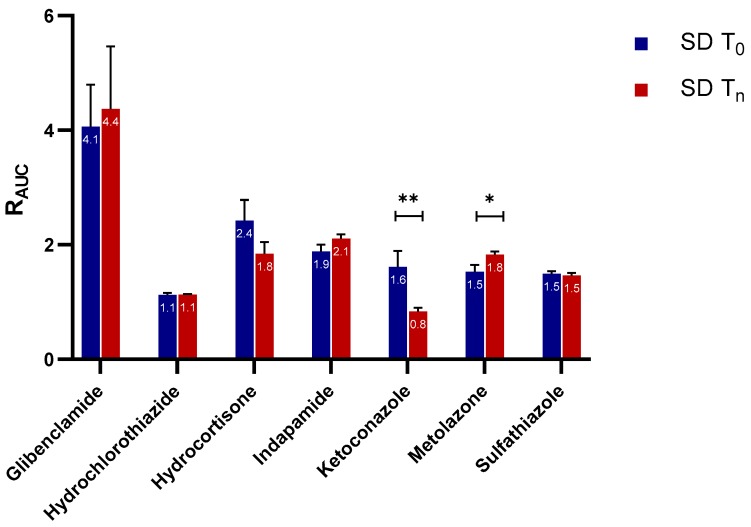
The area under the curve (AUC) ratio (R_AUC_) of the fresh and aged and/or crystallized spray-dried APIs at tenfold supersaturation ratios. The freshly spray-dried samples are represented as SD T_0_, whereas the crystallized and/or aged samples are denoted SD T_n_ since each of the APIs crystallized at different time points (see Table 1). At a 95% confidence interval, a *p*-value of <0.05 is considered statistically significant. The mean ± standard deviation values of three replicates are shown. Here, *p* < 0.05 and *p* < 0.01 are denoted with * and **, respectively.

For most of the spray-dried samples, no clear differences could be extracted from the dissolution profiles of the fresh versus the aged and/or the crystallized forms (Figure 1). We therefore analyzed the data further with statistical analyses on the C_max,app_ and AUC ratio (denoted by R_Cmax,app_ and R_AUC_, respectively) of both sample types for each API (Figure 2 and Figure 3). There was no significant difference in the R_Cmax,app_ of the fresh and the aged and/or crystallized spray-dried API samples except for ketoconazole, for which the R_Cmax,app_ decreased by 50% in the completely crystallized spray-dried sample (Figure 2). The small amounts (6% and 11%) of crystallinity present in the glibenclamide and hydrocortisone samples did not seem to have a significant impact on their C_max,app_. The fresh spray-dried hydrocortisone, hydrochlorothiazide, and sulfathiazole were 100% amorphous. It is therefore noteworthy that their R_Cmax,app_ values were similar to those of their completely crystallized forms after storage at 75% RH.

Similarly, the extent of supersaturation—indicated by the R_AUC_ of these samples—did not seem to be negatively impacted at a significant level by the aging and/or crystallization of the spray-dried samples (Figure 3). Only one of the seven APIs, ketoconazole, showed a major drop in the R_AUC_ ratio (i.e., from 1.6 to 0.8), which is in good agreement with the reduction in C_max_ ratio shown in Figure 2. Metolazone demonstrated a statistically significant increase in R_AUC_ in the aged spray-dried sample compared to the fresh one. A similar trend, although not statistically significant, was also noted for aged indapamide and partially crystallized glibenclamide. The same APIs showed a similar trend in their R_Cmax,app_ values for the aged and/or partially crystallized forms. One explanation may be a small amount of adsorption of water on the surface of the particles. It has been reported that a small amount of water adsorption can potentially have a positive effect on powders by eliminating interparticle electrostatic charges and irregularities [22,23]. This in turn reduces the tendency of the powder to agglomerate upon contact with water during wetting and dissolution; such a formation hinders dissolution by decreasing the effective surface area accessible by water during the process. The reduction in the stickiness and electrostatic nature of the powders of these APIs was indeed visible during our own experimental studies. In contrast, there were no significant differences in the R_AUC_ values of the fresh and crystallized samples of hydrocortisone, hydrochlorothiazide, and sulfathiazole. It can also be inferred from Figure 2 and Figure 3 that glibenclamide had the highest supersaturation potential among the APIs, for which the R_Cmax,app_ and R_AUC_ were approximately 7 and 4 times higher than the crystalline counterparts, respectively. The other APIs attained R_Cmax,app_ and R_AUC_ values only less than or about 1 to 3 times those of the crystalline forms.

**Figure 4:**
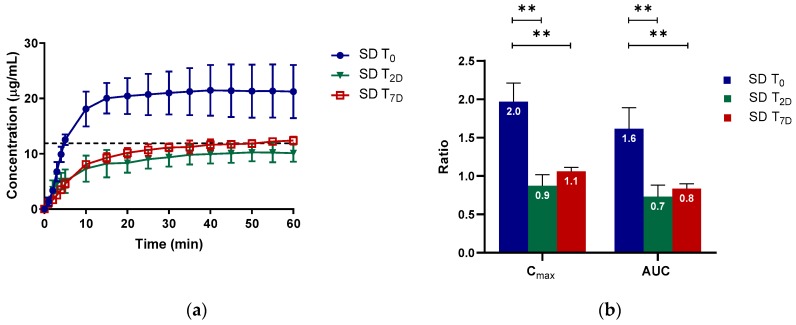
The (**a**) concentration–time profile and (**b**) C_max,app_ and AUC ratios (R_Cmax,app_ and R_AUC_) of fresh (SD T_0_), 2-day (SD T_2D_; 23% crystalline), and 7-day (SD T_7D_; completely crystalline) spray-dried ketoconazole stored at 75% RH. The mean ± standard deviation values of three replicates are shown. *p* < 0.01 is denoted **.

It was intriguing to observe that only ketoconazole was significantly compromised in its R_Cmax,app_ and R_AUC_ upon storage. To investigate the extent to which solid crystallization of ketoconazole would compromise its supersaturation potential, we compared the fresh (T_0_) and completely crystallized (T_7D_) sample with a 2-day sample (T_2D_) with an estimated crystalline content of 23%. Ketoconazole SD T_0_ had a higher supersaturation performance than both ketoconazole SD T_2D_ and ketoconazole SD T_7D_ (Figure 4a). Similar to the completely crystallized sample, the C_max_ and AUC ratios decreased by 50% for the 2-day-old sample containing 23% crystallinity (Figure 4b). Thus, for spray-dried ketoconazole, 23% crystalline content could compromise the supersaturation potential tremendously, jeopardizing the solubility enhancement from amorphization.

#### 2.2.2. Roles of Crystallization Pathways, Polymorphs, and Impurities on the Supersaturation and/or Kinetic Solubility of Spray-Dried Solids

Three of the seven APIs (hydrocortisone, hydrochlorothiazide, and sulfathiazole) displayed what seemed like a stable supersaturation although they were completely crystalline after storage at 75% RH for one day. Hence, the supersaturation was not a result of the amorphous nature of the spray-dried solid sample. We concluded that the dissolution and supersaturation behaviors were similar for the fresh and completely crystallized samples. No “spring” effect of supersaturation was observed even with the fresh samples of these APIs. It may be that the same crystallization pathway takes place in the solid amorphous particle during dissolution as during storage at high humidity. Another explanation is that a metastable crystal form is precipitating from solution, and this form exhibits higher solubility than the original crystal form from which the spray-dried solid was produced.

In a pure system, sufficient supersaturation must be reached for crystallization to take place. Depending upon the level of supersaturation at which nucleation occurs, the nucleation can be either homogenous or heterogenous. Homogenous nucleation requires a higher degree of supersaturation than heterogenous nucleation. In homogenous nucleation, the nuclei form from the bulk solution. In contrast, in heterogeneous nucleation, the nuclei form on solid surfaces or substrates. For this to occur, the solute molecules must have some affinity for solid substrates. These solid substrates can be API particles, the wall of the vial, dust, the stirrer, etc. In addition, the presence of ions and molecules other than the API molecules in the solution may also affect the nucleation, crystal growth, and the types and properties of the crystal formed [24].

During dissolution of an amorphous solid, crystallization can also occur via two different mechanisms—solid-to-solid or solution-mediated [25,26,27,28,29]. Solid-to-solid transformation occurs upon contact of the amorphous solid with water. Here the rapid dissolution of the solid induces a high local concentration of API solutes on the solid surface, which drives crystallization to occur on the surface of the particles. If the crystallization rate is considerably higher than the dissolution rate, the dissolution profile resembles the crystalline dissolution strongly. This suggests that there is minimal attainment and there is a loss of the amorphous solubility advantage. In contrast, solution-mediated crystallization occurs following supersaturation [24,30]. It encompasses three major steps: (i) dissolution of the amorphous (or any metastable) solid; (ii) nucleation of molecular units in the solution to form a more stable solid form; and (iii) growth of the stable form. These processes govern the concentration achieved during dissolution of an amorphous (or any metastable) form [24,31]. In most cases, one of these mechanisms predominates over the other, although in some cases both may take place to an equally great extent.

In addition to the crystallization mechanism, the environment to which the amorphous solid is exposed during the dissolution (i.e., temperature, agitation, pH, impurities, etc.) may influence the thermodynamics and kinetics of the transformation process [24,32,33,34]. Under some conditions, a metastable polymorph or a combination of different metastable polymorphs of the crystal may occur, giving rise to a higher solubility than the solubility of the stable polymorph.

Finally, the size of a crystal may also have an effect of the solubility profile of an API. It has been reported that a particle size of approximately ≤100 nm can increase the solubility of a given compound [35]. However, an increase in solubility and the generation of concentration–time profiles that resemble a stable supersaturation were observed only for three of the seven APIs (i.e., hydrochlorothiazide, hydrocortisone, and sulfathiazole). As such, it is unlikely that crystal size would contribute to the increased solubility, taking into consideration that all seven APIs were subjected to the exact same conditions during the supersaturation experiments.

**Figure 5:**
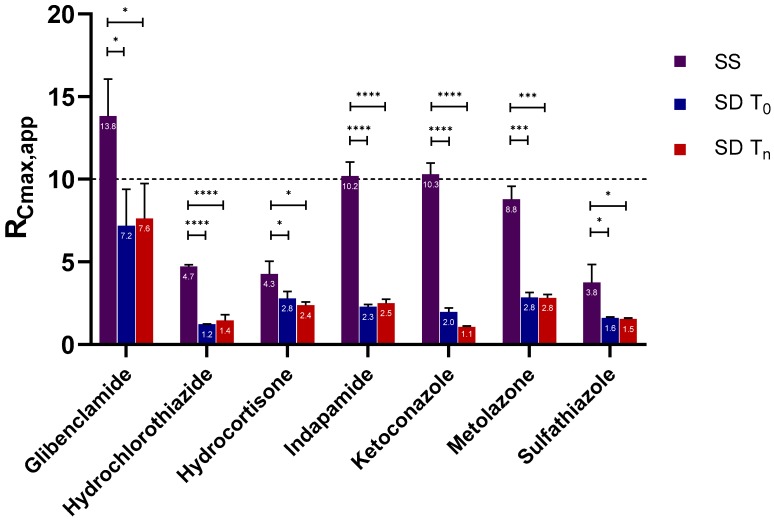
The apparent C_max,app_ ratio (R_Cmax_,_app_) of the solvent shift for fresh and aged and/or crystalline spray-dried APIs at tenfold supersaturation ratios. The solvent shift is represented as SS, the freshly spray-dried samples as SD T_0_, and the crystallized and/or aged samples as SD T_n_, since each of the APIs crystallized at different time points (see Table 1). At a 95% confidence interval, a *p-*value of <0.05 is considered statistically significant. The mean ± standard deviation values of three replicates are shown. The following statistics are described: *p* < 0.05 is denoted *, *p* < 0.001 is denoted ***, *p* < 0.0001 is deonted ****.

To investigate the possible contribution of any of these factors, supersaturation was generated using a solvent shift approach. In particular, we wanted to look at hydrocortisone, hydrochlorothiazide, and sulfathiazole. Similarly, tenfold supersaturation ratios were tested. Via the solvent shift method, the dissolution step is avoided, as the supersaturation is produced from a concentrated solution of the API dissolved in DMSO. The solvent shift method probes the crystallization mechanism of the APIs based on their supersaturation profiles. In addition, solid-state analysis was also performed because it gives more insight into possible polymorphic changes during the dissolution and crystallization process. Again, the R_Cmax,app_ was assessed and compared to those of the spray-dried samples. The results are displayed in Figure 5.

As seen in Figure 5, all APIs reached a higher level of R_Cmax,app_ when supersaturation was generated from a stock solution than when it was generated from dissolving spray-dried solids. The presence of undissolved spray-dried solid had a tremendously negative effect on the ability of the compounds to reach the full R_Cmax,app_ level. The figure shows two distinguishable patterns in R_Cmax,app_ attainment. On the one hand, four APIs—indapamide, metolazone, glibenclamide, and ketoconazole—succeeded in reaching the highest possible R_Cmax,app_ (i.e., 10). On the other hand, the other three compounds had immensely lowered (fivefold) R_Cmax,app_ values compared to their crystalline solubility. These observations may be related to the nature of the nucleation process—it being a result of homogenous or heterogeneous nucleation. Crystallization of glibenclamide, indapamide, ketoconazole, and metolazone from a relatively higher degree of supersaturation suggests that they nucleated homogenously from the bulk solution. Additionally, their supersaturation at these higher levels implies that they predominantly underwent solution-mediated crystallization. The same mechanism of crystallization upon dissolution of indapamide, metolazone, and glibenclamide has been previously reported [29]. In contrast, the hydrocortisone, hydrochlorothiazide, and sulfathiazole nucleated heterogeneously. For these compounds, the large number of particles and the resulting large solid (amorphous) surface area available to nucleate on causes their nucleation to be particle-associated, or solid-to-solid mediated, during dissolution.

**Figure 6:**
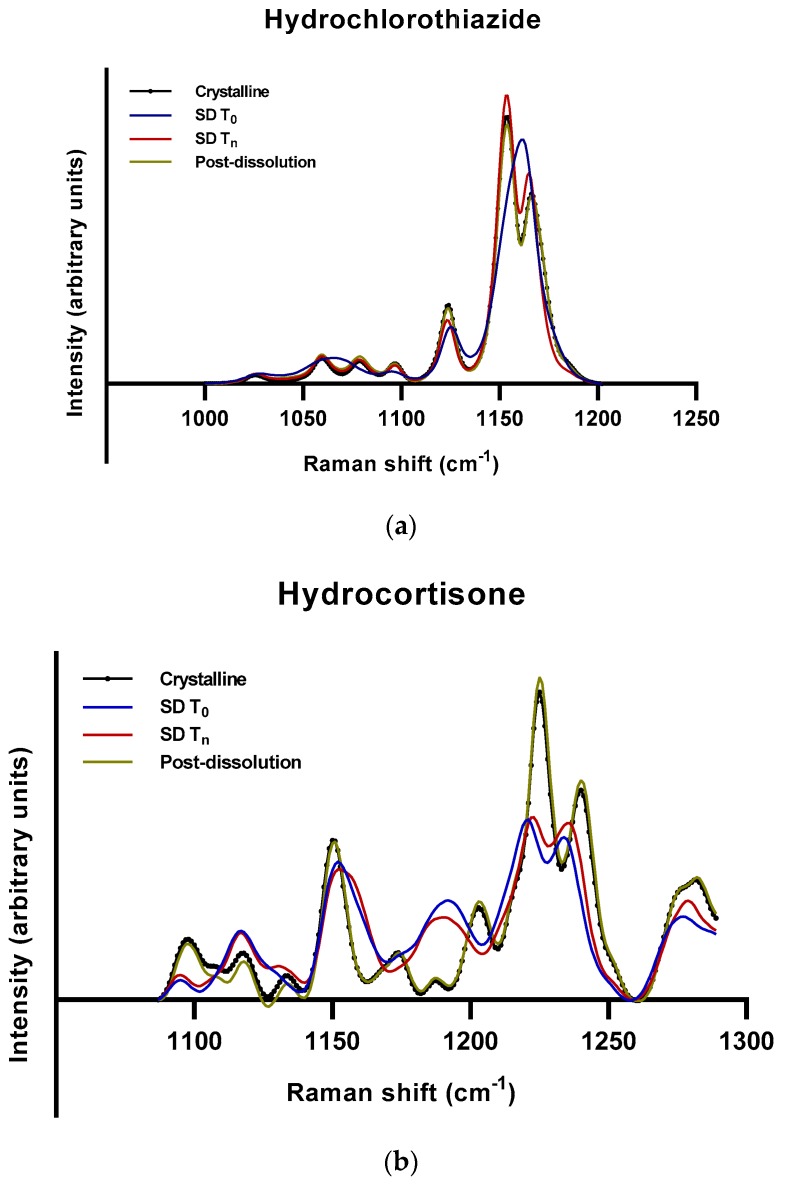

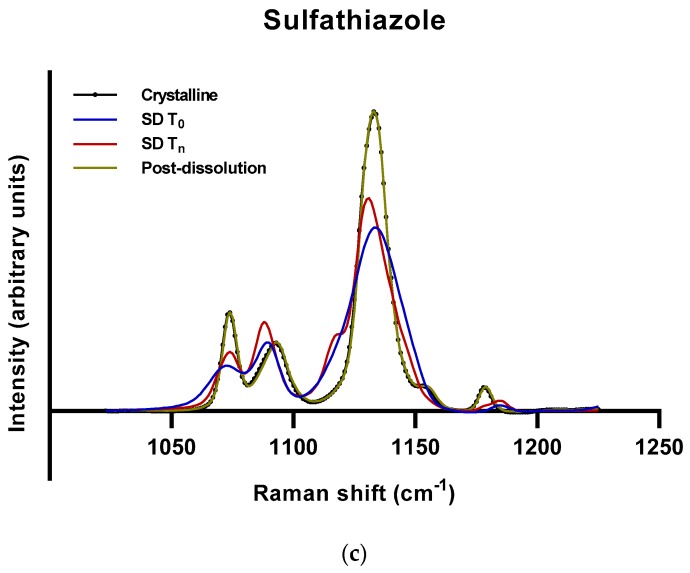
Raman spectra of different samples of (**a**) hydrochlorothiazide, (**b**) hydrocortisone, and (**c**) sulfathiazole. The samples represented are unprocessed crystalline (black circle); fresh spray-dried/complete amorphous, SD T_0_ (blue); spray-dried crystallized at different time points at 75% RH, SD T_n_ as indicated in Table 1 (red); and post-dissolution of SD T_0_ (olive).

To rule out the role of experimental variability in the dissolution and/or supersaturation profiles of hydrocortisone, hydrochlorothiazide, and sulfathiazole, additional solid-state analyses were performed on the remaining solids collected after the dissolution experiments. These remaining solids were subjected mainly to Raman spectroscopy and, when necessary, to differential scanning calorimetry. The Raman spectra of these three APIs indicate that they crystallized to the original crystalline forms upon dissolution (Figure 6a–c). What is also striking is that hydrocortisone—which existed as an amorphous–crystalline mixture after 168 days of storage at 75% RH—became crystalline after dissolution. Hydrocortisone produced a concentration–time profile which appeared like stable supersaturation with a concentration above its crystalline solubility (Figure 1). This indicates that the stable supersaturation was not obtained from the amorphous nature of the sample. Even though it is not very clear from the Raman spectra, the most plausible explanation is the possible formation of a metastable polymorph. Sulfathiazole, in contrast, crystallized as a different polymorph when exposed to 75% RH.

**Figure 7:**
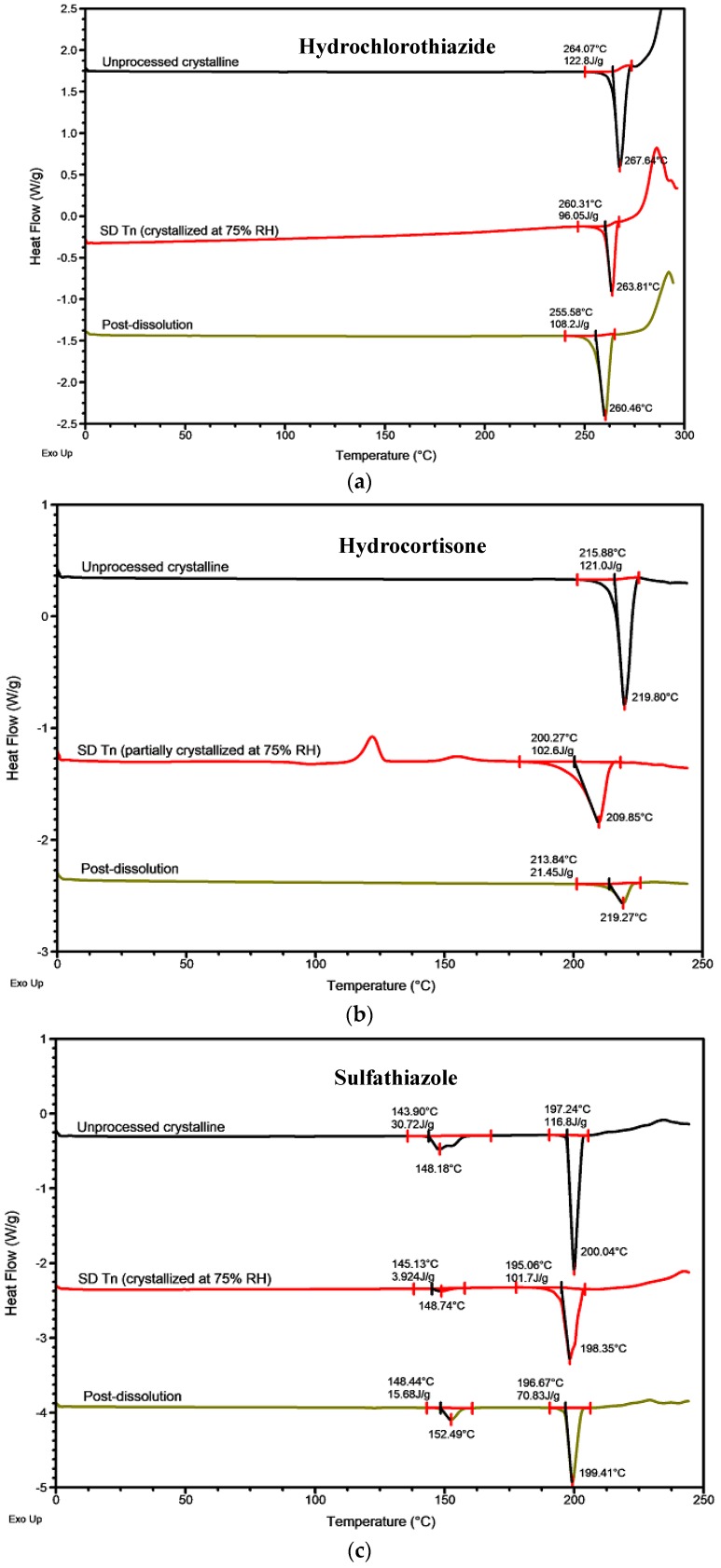
The differential scanning calorimetry thermograms of (**a**) hydrochlorothiazide, (**b**) hydrocortisone, and (**c**) sulfathiazole. For each API, the thermograms of unprocessed crystalline API, spray-dried samples stored at 75% RH, SD T_n_ (partially, or completely crystallized), and post-dissolution solid samples are shown. A representative of duplicate runs is shown for each sample type.

The differential scanning calorimetry thermograms of these three APIs displayed slightly different onsets of melting and heat of fusion, which could be an indication of different individual polymorph(s) or a mixture formed upon crystallization at 75% RH (Figure 7a–c). For instance, the somewhat lower onset of melting (260 °C) of hydrochlorothiazide suggests a possible formation of a crystal with lower crystal binding strength (Figure 7a). An even lower onset of melting (255 °C) was detected from the post-dissolution sample. Similarly, hydrocortisone showed a lower onset of melting and heat of fusion for both the partially crystallized spray-dried sample stored at 75% RH and the post-dissolution sample compared to the unprocessed crystalline ones. This again may be attributed to the formation of less stable polymorph(s) (Figure 7b).

The differential scanning calorimetry thermograms of sulfathiazole appeared slightly different from those of hydrochlorothiazide and hydrocortisone. The unprocessed crystalline sulfathiazole exists as a mixture of at least three polymorphs with melting occurring between 143 °C and 197 °C. Crystallization at 75% RH produced a slightly different mixture from the unprocessed crystalline one. One of the overlapping melting peaks disappeared, suggesting that only one of those polymorphs, with an onset of melting around 145 °C, remained after the crystallization. The melting peak with a higher temperature also broadened, leading to lower onset of melting and heat of fusion. After dissolution, again, a different mixture of polymorphs was produced. The first melting peak started at approximately 148 °C, marginally higher than for the 75% RH sample. The second melting peak very closely resembled the unprocessed crystalline sulfathiazole (~197 °C) except that the peak was broader, which indicates weaker crystal strength. This interpretation is supported by the lower heat of fusion of the second melting peak (Figure 7c). Together, these results could partly explain why the solubility levels of the fresh and spray-dried samples of these APIs were relatively higher than that of the unprocessed crystalline form. Even though hydrochlorothiazide, hydrocortisone, and sulfathiazole have been reported to have several different crystal forms [36,37,38], the exact crystal forms corresponding to the ones observed in our study could not be clearly identified.

Besides the formation of metastable polymorphs, the observed differences in the thermal properties (i.e., onset of melting and heat of fusion) of these three APIs may be due to the formation of the same polymorph with a higher degree of disorder and weaker crystal strength. A crystal structure with weaker strength can be produced when crystallization occurs in impure solution via the inclusion of impurities into the crystal structure. These impurities can originate from the dissolution medium (ions from the salts), remaining organic solvent for e.g., the amorphization or impurities present already in the API as supplied from the manufacturer. The roles of the solvent/solution where crystallization occurs and impurities in influencing the crystallization process and the resulting crystals’ properties have been well reported and described in the literature [39,40,41,42].

#### 2.2.3. Crystallization Rate Constant (k) and Crystallization Kinetics

**Figure 8:**
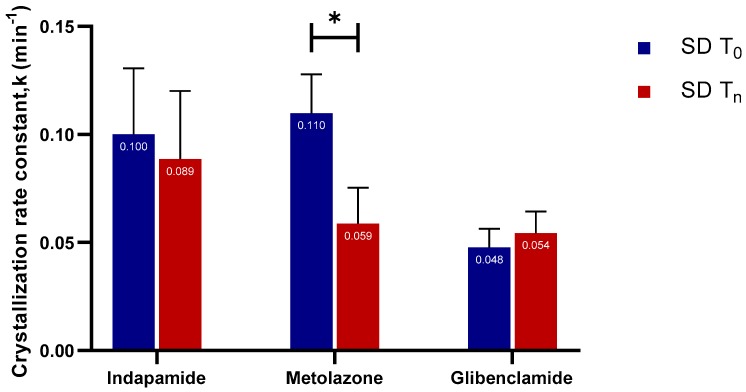
The crystallization rate constant, k (min^−1^), of fresh and aged and/or crystallized spray-dried indapamide, metolazone, and glibenclamide. *p* < 0.05 is denoted *.

The crystallization rate constant (k) reflects the rate at which crystallization progresses. Slow crystallization process is associated with a small rate constant value, while a large rate constant is linked to rapid crystallization. Typically, as nucleation and crystal growth proceed, the overall supersaturation decreases. Accordingly, the kinetics of nucleation and crystal growth slow down as the system approaches equilibrium. As that happens, thermodynamic factors begin to dominate over the kinetic ones [24]. Out of the seven APIs, the crystallization rate constant could only be calculated for indapamide, metolazone, and glibenclamide (Figure 8).

This is because the concentration–time profiles of the hydrocortisone, hydrochlorothiazide, ketoconazole, and sulfathiazole did not show the typical “spring” characteristic associated with a supersaturating system; thus, there was no decline in concentration after C_max,app_. Only the crystallization rate constant of the aged, spray-dried metolazone showed a significant decrease when compared to its fresh counterpart. For indapamide and glibenclamide, there were no significant differences. This augments the fact that the small amount of crystallinity in glibenclamide did not affect the crystallization rate.

## 3. Materials and Methods

### 3.1. Materials

Glibenclamide and hydrocortisone (purity ≥98%) were purchased from Sigma-Aldrich (Munich, Germany). Sulfathiazole (purity ≥98%) was procured from Fluka Analytical, Sigma (Germany). Indapamide (purity >97%) was purchased from TCI Europe (Zwijndrecht, Belgium). Metolazone was acquired from APIChem Technology Co (Hangzhou, China), and hydrochlorothiazide and ketoconazole were purchased from TRC (North York, Canada); these were of analytical grade.

### 3.2. Preparation of Amorphous APIs and Physical Stability Study

The amorphous solids were prepared by spray-drying. In short, solutions containing the APIs of interest were prepared by dissolving the crystalline powder in either ethanol, acetone, or a mixture of 90:10 (% *w*/*w*) ethanol/acetone prior to spray-drying. Any unwanted debris and particulate contaminants were removed by filtration (0.45 µm pore size). Thereafter, the solutions were spray-dried using a Büchi B-290-Mini Spray Dryer with an inert loop (Büchi Laboratoriums, Flawil, Switzerland) according to the processing parameters previously described by Edueng et al. [29]. After spray-drying, the collected solids were divided and kept in two different wide-mouth aluminum containers. These open containers were placed in a vacuumed desiccator overnight to remove any residual solvent. The vacuum-dried solids were subsequently characterized with different solid-state analytical methods, studied for their supersaturation behavior, and stored in the stability chamber for 168 days (6 months). Following the overnight vacuum-drying, the spray-dried materials were placed in a stability chamber with a 75% relative humidity level and constant temperature of 25 °C. The samples were stored in open wide-mouth aluminum containers. Samples were withdrawn from the stability chamber at 1, 2, 7, 14, 28, 84, and 168 day(s) for the solid-state analyses. These analyses monitored the physical stability of the amorphous API, and the supersaturation study investigated the impact of solid-state changes on the supersaturation potential and crystallization kinetics. The preparation of these materials and the physical stability protocol are described in detail in Edueng et al. [43].

### 3.3. Solid-State Analysis

#### 3.3.1. Powder X-ray Diffraction (PXRD)

A Twin-Twin (Bruker, Coventry, UK) measuring in Bragg–Brentano geometry and equipped with a sample rotator was used to collect the X-ray diffractograms of the unprocessed crystalline and freshly spray-dried APIs. The solid samples were gently ground with a mortar and pestle when the powder was too coarse. Thereafter, they were placed on silicon sample holders which rotated at 40 rpm during the measurement. The X-ray source was a rotating copper anode with an accelerating voltage of 40 kV and current of 40 mA. The emitted Cu Kα radiation (λ = 1.54 Å) was used in the measurements. A diffraction pattern in the 2Ө range between 5° and 40° was collected at a scanning step of 0.01° and 0.3 s exposure time at each step. Primary and secondary divergence slits of 0.40 and 2.48 mm, respectively, were used. Since this technique provides a more direct interpretation regarding the amorphous and/or crystalline content of samples, it was used to ensure that the spray-dried APIs were fully amorphous prior to the initiation of other analyses. Based on this information, the reference Raman spectra for the fully amorphous and fully crystalline samples of each compound could be established.

#### 3.3.2. Modulated Differential Scanning Calorimetry (MDSC)

Modulated differential scanning calorimetry (MDSC) was performed to identify and monitor the solid-state form changes of the solids (amorphous, crystalline, or a mixture of both) and to determine the temperatures of glass transition (T_g_), crystallization (T_c_), and melting (T_m_). Approximately 1–5 mg of sample was weighed and transferred into standard aluminum pans. The lids were pin-holed to allow evaporation of any solvent or moisture. Depending on the T_g_, the samples were equilibrated at either −70 °C or 0 °C to allow for at least a 20 °C baseline prior to the T_g_ measurement, followed by heating up to 20–30 °C above the T_m_ at 3 °C/min and a temperature modulation of ±1 °C every 60 s. Analyses and integrations of the thermograms were performed using TA Instruments Universal Analysis 2000 for Windows (Version 4.5A, Sollentuna, Sweden) software.

#### 3.3.3. Raman Spectroscopy

In addition to MDSC, the samples were examined by Raman spectroscopy using a Rxn-2 Hybrid Raman Spectrometer (Kaiser Optical System Inc., Ann Arbor, MI, USA). These samples included (i) the solid-state form of the unprocessed crystalline materials and freshly spray-dried solids; (ii) any solid-state transformation of the spray-dried solids after exposure to 75% RH at designated time points; and (iii) solids collected after supersaturation studies (when necessary). The Raman spectrometer, coupled with a fiber-optic PhAT probe, has a laser wavelength of 785 nm and maximum laser power of 400 mV. For the analysis, about 5–20 mg of samples was placed on an aluminum sample holder, after which the spectra in the wavenumber range of 100–1890 cm^−1^ were collected. These spectra were then processed to allow semi-quantitative analysis of the crystallinity of the samples, as described in Section 3.4.

### 3.4. Calculation of Amorphous Content Using Raman Spectra

The changes in the amorphous and/or crystalline content of the spray-dried samples stored at 75% RH were calculated via semi-quantitative analysis of the Raman spectra. The same method as described in Edueng et al. [43] was used, and this should be referred to for details. In short, a wavenumber region with large differences between spray-dried and crystalline was selected for the analyses. The spectra were background corrected assuming a straight baseline between the endpoints of the selected region and normalized by the summed intensity of the background-corrected spectrum. By least square fit of the weighted sum of the crystalline and amorphous (freshly spray-dried sample) spectra to the 75% RH stored sample spectrum, semi-quantification of the crystalline and amorphous contents for the stored sample was obtained as the weights (*f_CR_* and *f_SD_*). With this method, a limit of detection (LOD) of 2% was achieved.

### 3.5. In Vitro Supersaturation under Non-sink Conditions

The in vitro supersaturation studies were performed on freshly spray-dried samples and the stored spray-dried ones that (i) had remained completely amorphous; (ii) formed a mixture of amorphous and crystalline; or (iii) completely crystallized before 168 days. For samples that remained completely amorphous or formed a mixture of amorphous and crystalline after 168 days of storage, the in vitro supersaturation study was carried out on day 168. On the other hand, samples that completely crystallized before 168 days were subjected to the supersaturation study on the day when complete crystallinity was observed from the solid-state analyses. The protocol is described in Edueng et al. [29]. In short, the experiment was initiated by constructing a standard curve (R^2^ ≥ 0.97) covering the possible concentrations attained from the supersaturation. An amount of solid equivalent to tenfold of the apparent crystalline solubility was added into 3 mL phosphate buffer at a pH of 6.5 to create a non-sink condition and generate a tenfold supersaturation ratio. For this purpose, a small-scale dissolution apparatus, the µDISS Profiler (pION Inc., Billerica, MA, USA), was used. The temperature and stirring rate were maintained at 37 °C and 100 rpm, respectively, and the supersaturation behavior was monitored for 60 min. The experiments were conducted in triplicate for each sample type.

### 3.6. In Vitro Supersaturation Determined by a Solvent Shift Method

To probe the predominant crystallization pathway of the APIs in solution, we also assayed the level of supersaturation using a solvent shift method. This method bypasses the dissolution process as the supersaturation is generated by introducing the API in dissolved form (as a dimethyl sulfoxide stock solution). The solvent shift method also allows a comparison of the supersaturation profiles of the spray-dried APIs.

#### 3.6.1. Stock Solution Preparation

The stock solutions for each of the drugs were prepared by dissolving the compounds in 100% dimethyl sulfoxide (DMSO). The concentration of the stock solution to be produced was determined by the apparent crystalline solubility of the API. We aimed for concentrations close to the maximum solubility of the compound in DMSO to minimize the volume of the solution when creating the tenfold supersaturation ratio. To minimize the effect of the DMSO on drug solubility, the total concentration of DMSO solution in the dissolution medium was kept at ≤2%.

#### 3.6.2. Solvent Shift Experiment

Similar to the supersaturation study in the previous section, a calibration curve was established before the solvent shift experiment. Thereafter, the supersaturation studies were performed under non-sink conditions using the µDiss Profiler. As in the supersaturation study described in Section 3.5, 3 mL of phosphate buffer at pH 6.5 was used as the dissolution medium, the temperature was set to 37 °C, and a stirring rate of 100 rpm was used. The volume of standard solution required to achieve the desired supersaturation was dependent on the concentration of the prepared DMSO solution. The level of supersaturation was measured at tenfold the crystalline solubility. The supersaturation for each API was measured in triplicate and followed for 60 min. The results are expressed as mean ± standard deviation (SD).

### 3.7. Calculation of the Apparent Maximum Concentration, Area under the Curve, and Crystallization Rate Constant

**Figure 9:**
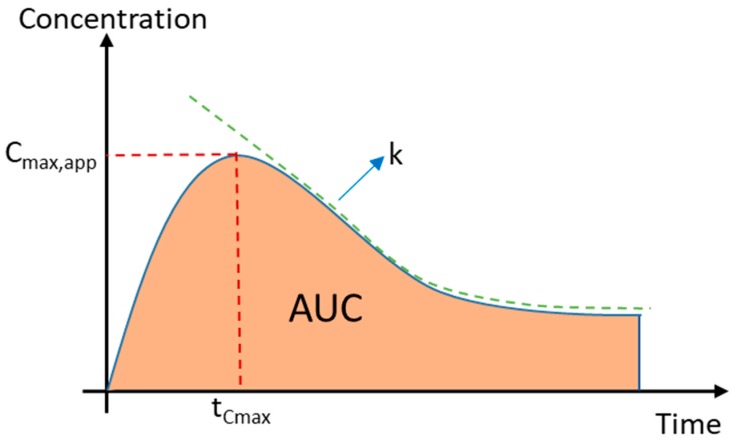
Concentration–time profile of the supersaturated system showing the apparent maximum concentration (C_max,app_), area under the curve (AUC), and crystallization rate constant (k).

The effects of physical aging and/or crystallization upon storage on the performance at tenfold supersaturation were assessed on the basis of the following parameters: (i) the apparent maximum concentration achieved (C_max,app_); (ii) the area under the curve (AUC); and (iii) the crystallization rate constant (k) (see Figure 9). C_max,app_ refers to the maximum supersaturation concentration attained by dissolving amorphous solid in the medium before a decline in concentration was observed. The extent of supersaturation obtained is described by the AUC, which takes into consideration both the time and concentration of the supersaturated system over the course of the study (i.e., 60 min). The crystallization rate constant (k) defines the rate at which a decrease in concentration was observed. It gives an indication of the crystallization rate upon dissolution and is expressed as the reciprocal of the *x*-axis time units.

These three parameters were determined using GraphPad Prism version 8.1.0 for Windows (GraphPad Software, San Diego, CA, USA). C_max,app_ was obtained by determining descriptive statistics of the tabulated dissolution data. It corresponds to the maximum value or highest point in the dissolution data (referred to as “maximum” in the Graphpad software). The AUC was computed based on the trapezoidal rule. The C_max,app_ and AUC of the fresh and the aged/crystallized spray-dried samples were divided by the C_max,app_ and AUC of the crystalline samples to obtain the ratios of both parameters (see Equations (1) and (2)). The ratios were then referred to as R_Cmax,app_ and R_AUC_, respectively. The crystallization was approximated following first-order kinetics, leading to Equation (3). The k value was determined by performing a nonlinear curve fitting of Equation (3) from the time point of C_max,app_ of the supersaturation profiles of the samples using the same software.

(1) $$R_{\mathrm{Cmax},\mathrm{app}}=\frac{C_{\max,\mathrm{SD}}}{C_{\max,\mathrm{cryst}}}$$

(2) $$R_{\mathrm{AUC}}=\frac{\mathrm{AU}C_{\mathrm{SD}}}{\mathrm{AU}C_{\mathrm{cryst}}}$$

(3) $$C\left(t\right)=\left(C_{0}-C_{\infty }\right)\cdot e^{-k\cdot t}+C_{\infty }$$

In Equation (3), C_0_ is the fitted concentration at t = 0, C_∞_ is the estimated concentration at infinite time, and k is the rate constant. In our study, the concentration is expressed in µg/mL while k is expressed in min^−1^.

### 3.8. Statistical Analysis

For all sample types, the values are reported as mean ± standard deviation (SD). The standard deviations were calculated from values of three replicates (n = 3) at a 95% confidence interval. To test for the statistical significance of any observed differences in the maximum R_Cmax_,_app_ R_AUC,_ and k, an unpaired t-test was performed on dissolution data of the fresh and aged/crystallized samples. GraphPad Prism version 8.1.0 for Windows was used. At a 95% confidence interval, a *p-*value of <0.05 was considered statistically significant.

## 4. Conclusions

For most of our model APIs, the overall supersaturation potential was affected to a low extent by aging and/or crystallization under humid conditions for 168 days. Glibenclamide and hydrocortisone—with crystalline contents of 6% and 11%, respectively—showed no significant difference in their apparent maximum concentration, area under the curve, and crystallization rate constant. In contrast, the two-day old ketoconazole—with 23% crystallinity—totally lost its supersaturation potential. Its apparent maximum concentration and area under the curve were similar to those of the original crystalline form. A few APIs, like indapamide and metolazone, exhibited excellent physical stability but crystallized rapidly upon dissolution.

This study highlights that crystallization pathways dictate the supersaturation potential of the API. Amorphization of hydrocortisone, hydrochlorothiazide, and sulfathiazole, for instance, did not lead to an increased amount of dissolved material due to their rapid crystallization. Instead, phase transformation during dissolution gave rise to the formation of a metastable polymorph or a mixture of several polymorphs which may be mistaken as stable supersaturation contributed by dissolution of the amorphous solid. However, the formation of the same polymorph as the original crystalline sample supplied by the manufacturer, with lower crystal strength due to the inclusion of impurities, cannot be entirely excluded as a contributing factor in the higher observed solubility of these three APIs.

Taken together, our results signify that the crystallization (and its pathways) during dissolution deserves greater attention and better understanding if one is to select a proper crystallization inhibitor/stabilizer for stabilizing an API. A good, solid-state crystallization inhibitor/stabilizer might not work as well in solution and vice versa. Therefore, understanding which crystallization pathway to inhibit is necessary to guide the formulation selection process.

## Figures and Tables

**Table 1:** Stability profile of spray-dried amorphous active pharmaceutical ingredients (APIs) upon exposure to 75% RH at 25 °C for 168 days.

API	Time to Start Crystallization (Day)	Time to Complete Crystallization (Day)	Crystallization Initiation Propensity	Crystal Growth	Estimated Amorphous Content at Day 168 (%)	Stability for 168 Days at 75% RH
Indapamide	>168	>168	Low	n.d.	100	High
Metolazone	>168	>168	Low	n.d.	100	High
Glibenclamide	1	>168	High	Slow	94	Moderate
Hydrocortisone	1	>168	High	Slow	89	Moderate
Ketoconazole	1	7	High	Rapid	0	Low
Hydrochlorothiazide	1	1	High	Rapid	0	Low
Sulfathiazole	1	1	High	Rapid	0	Low

n.d. = not determinable.
